# A Qualitative Study to Develop a Privacy and Nondiscrimination Best Practice Framework for Personalized Wellness Programs

**DOI:** 10.3390/jpm10040264

**Published:** 2020-12-03

**Authors:** Rachele M. Hendricks-Sturrup, Kathy L. Cerminara, Christine Y. Lu

**Affiliations:** 1 Department of Population Medicine, Harvard Pilgrim Health Care Institute, Harvard Medical School, Boston, MA 02215, USA; christine_lu@harvardpilgrim.org; 2 Shepard Broad College of Law, Nova Southeastern University, Fort Lauderdale, FL 33314, USA; cerminar@nova.edu

**Keywords:** wellness programs, mobile applications, direct-to-consumer screening and testing, health policy, personalized medicine

## Abstract

Employers in the United States (US) increasingly offer personalized wellness products as a workplace benefit. In doing so, those employers must be cognizant of not only US law but also European Union (EU) law to the extent that the EU law applies to European immigrants or guest workers in the US. To the extent that wellness programs are implemented in either public health or employment contexts within the US and/or EU, sponsors of these programs can partner with direct-to-consumer (DTC) genetic testing companies and other digital health companies to generate, collect, and process sensitive health information that are loosely or partially regulated from a privacy and nondiscrimination standpoint. Balancing claims about the benefits of wellness programs are concerns about employee health privacy and discrimination and the current unregulated nature of consumer health data. We qualitatively explored the concerns and opinions of public and legislative stakeholders in the US to determine key themes and develop privacy and nondiscrimination best practices. Key themes emerged as promoting a culture of trust and wellness. Best practices within these themes were: (1) have transparent and prominent data standards and practices, (2) uphold employee privacy and nondiscrimination standards, (3) remove penalties associated with biometric outcomes and nondisclosure of sensitive health information, (4) reward healthy behavior regardless of biometric outcomes, and (5) make program benefits accessible regardless of personal status. Employers, DTC genetic testing companies, policymakers, and stakeholders broadly should consider these themes and best practices in the current absence of broad regulations on nondiscriminatory workplace wellness programs.

## 1. Introduction

Employer-sponsored wellness programs are popular in the United States (US) and are a welcomed concept by many given their focus on promoting health and well-being in the workplace. In the US, where health insurance coverage is heavily subsidized by employers who have an interest health insurance cost savings through promoting healthy behavior, over 157 million employers subsidized health insurance coverage across the total US population in 2018 [1]. An annual survey conducted between January and July 2019 among 2012 employers or firms found that 31% of small firms and 60% of large firms surveyed offered weight loss programs to their employees [2]. Additionally, during this same period, 39% of small firms and 71% of large firms surveyed offered lifestyle or behavioral coaching programs [2]. Moreover, 41% of large firms surveyed incentivized their employees to participate in or complete wellness or health promotion programs [2]. The Patient Protection and Affordable Care Act (ACA) amended existing federal privacy and nondiscrimination policies to make it easier for small and large employers to offer wellness program benefits and incentives. Wellness programs under the ACA are also supported by amendments to the Genetic Information and Nondiscrimination Act (GINA), the Americans with Disabilities Act (ADA), and the Health Insurance Portability and Accountability Act (HIPAA), plus accompanying regulations. To the extent that US employers collect data from European immigrants or guest workers in the US, the EU’s General Data Protection Regulation (GDPR) may govern [3]. Additionally, and in contrast to the US, the EU considers wellness programs as a broader public health initiative versus one situated only within workplace settings [4]. The EU has yet to fully consider or describe, however, a nuanced approach to uphold privacy and nondiscrimination protections for personal health or genetic data collected under broader, public wellness program initiatives.

Some studies show a possible benefit to wellness program participation among some employee populations, particularly around such programs’ potential to encourage healthy behaviors and reduce average medical claims payments [5,6,7]. However, the overall benefits of wellness programs, in terms of their ability to improve biometric outcomes (e.g., improved weight, blood pressure, waist circumference, body mass index, etc.), sustain healthy behaviors, and reduce overall health care expenditures, remain elusive. Still, US employers want to explore ways to engage their employees in corporate wellness programs to encourage healthy within and outside of the workplace, with the ultimate goal of reducing the employers’ overall health expenditures through better employee biometrics and/or health outcomes [8].

The concept of “personalized wellness” offers, for example, personalized wellness and nutrition advice based on diet, biomarker, and genotype information [9]. This concept has attracted employers in the US to partnerships with companies that offer employees direct-to-consumer (DTC) genetic testing services, consumer health wearables, and wellness apps, which collect sensitive health information to offer personalized health or wellness reports [8,10,11,12]. Countering claims about the benefits to these programs are concerns about health information privacy and coercion if wellness program incentives are tied to health insurance premium discounts (i.e., see *AARP v. EEOC*, 292 F. Supp. 3d 238, 243 [D.D.C. 2017]). For instance, the Equal Employment Opportunity Coalition (EEOC) in the US has determined that 30% insurance premium discounts might coerce employees into disclosing their sensitive health information to employers, DTC genetic testing companies, consumer wearable companies, and wellness apps in order to access health insurance premium discounts. None of these entities accessing sensitive employee health information are bound by specific health information privacy laws, even though they may collect, process, share, and take action based on the disclosure of sensitive health information [2,13,14,15,16,17,18,19]. Researchers recently reported that many individuals are willing to make “significant personal tradeoffs”, such as generating and sharing personal health and health behavior data, in exchange for monetary incentives like lowered insurance costs [20,21]. Therefore, preserving health information privacy by declining participation in employer-sponsored wellness programs may be less attractive (e.g., less affordable) for employees who likely desire ways to offset their health insurance costs.

Wellness program regulations under the ACA were vacated in January 2019 by a federal judge who decided that the then-current wellness program regulations failed to demonstrate “voluntariness” and meet nondiscrimination standards under GINA and the ADA (see *AARP v. EEOC*). As a result, there is an absence of federal regulatory guidance regarding nondiscriminatory workplace wellness programs. In late September 2019, the US Department of Health and Human Services announced an opportunity for states to apply and participate in a wellness program demonstration project that involves implementing nondiscriminatory health-contingent wellness programs that follow the provisions of section 2705(j) of the Public Health Service Act in the individual market [22]. 

In general, and beyond meaningful discourse and commentary, there is a dearth of qualitative evidence to inform policymaking processes regarding workplace wellness programs as they become more personalized though the use of consumer-generated data in both the US and EU. What is needed are best practices intended to define and address, through the development of an actionable framework, privacy and discrimination concerns within the context of personalized wellness programs. To address these gaps, we conducted a qualitative study to (1) define US public stakeholder concerns about privacy and discrimination following the disclosure of sensitive health information in employer-sponsored wellness programs, (2) discuss these concerns in a 1:1 fashion with US legislators and legislative staff to garner their suggestions to overcome these concerns, and (3) codify legislators’ and legislative staff’s suggestions as strategies that can be transformed into an actionable policy framework for personalized wellness program data governance.

## 2. Materials and Methods

### 2.1. Identification and Assessment of Public Stakeholder Concerns

An online search was conducted to identify public stakeholder concerns about employer access to genetic and health information and risk for discrimination under employer-sponsored wellness programs using permutated queries that included the following terms: wellness program, employer, discrimination, and genetic. As a starting point, a search was conducted initially in August 2018, and repeated in January 2019, within the Federal Register, which contains public comments received in response to proposed regulations. A Google search for transcripts of public testimony at federal legislative committee hearings was also conducted. Searches were also conducted in Westlaw, Lexis Nexis, and Bloomberg BNA to identify opinions, briefs, and pleadings filed in court cases citing violations of nondiscrimination laws (e.g., GINA and the ADA) and complaints about personal health or genetic information disclosure in employer-sponsored wellness programs after 2008, or after the year in which GINA became effective. Legal research access was made available on behalf of Nova Southeastern University Shepard Broad College of Law library. Court cases cited in public stakeholder comments within the Federal Register were also reviewed. Court case records (*n* = 36) and transcripts of public testimony from legislative hearings (*n* = 18) were uploaded to NVivo 11 software for qualitative analysis. Document titles were exported from the Federal Register to Microsoft Excel to generate a random sample of 259 substantive comments within documents in the Federal Register (sampled at a 95% confidence level, 5% margin of error, total number of documents = 788). R.M.H-S. analyzed substantive comments within Federal Register documents, court cases, and transcripts of public testimony using NVivo. We also carried out inductive data coding using the constant comparative analysis within the grounded theory approach [23] (to identify and develop themes). Qualitative assessment continued until thematic saturation was reached.

### 2.2. Interview Guide Development

Key concerns identified from the assessment of public comments, court case documents, and transcripts of public testimony were used to develop a written guide to conduct semi-structured interviews (in-person and phone) with US legislators and legislative staff. The final semi-structured interview guide contained 16 questions, 13 of which were open-ended, and was designed to be completed in roughly 25 min.

#### Legislator/Legislative Staff Interviews

To begin recruitment for interviews, R.M.H-S. contacted the legislators via email and telephone between March and May 2019, although recruitment was unsuccessful. In-person recruitment in Washington DC during June 2019 proved to be more successful, as a single recruitment meeting resulted in effective snowball sampling. Snowball sampling effectively allowed the participating legislators and legislative staff to invite their colleagues to participate in the study. Interviewees were given a brief explanation of the study rationale and purpose and their privacy rights as study participants, and were prompted to schedule a convenient time for the interview. Ten interviews were conducted by R.M.H-S. and audio-recorded with participants’ consent and transcribed verbatim. Standard content and thematic analysis procedures were used to generate initial coding categories using the interview questions as a starting point. Initial data coding was conducted using NVivo. A focused coding strategy was used to code comments in each transcript; R.M.H-S. read each transcript and created a coding tool. NVivo was then used to organize the data from the transcripts into categories based on codes and subcodes. A second coder was used to confirm agreement and interrater reliability using Microsoft Excel. Discrepancies in coding were resolved between the two coders and the main themes were outlined. Illuminating or example quotes within key themes and best practices were identified.

Demographic data were collected for each participant, including gender, congressional office, political party, and age (whether over 55 years). These data were collected or inferred during the interviews or from each legislator’s personal Web page. The Institutional Review Board at Harvard Pilgrim Health Care Institute approved this study.

## 3. Results

### 3.1. Assessment of Public Comments, Court Case Documents, and Transcripts of Public Testimony

**Figure 1:**
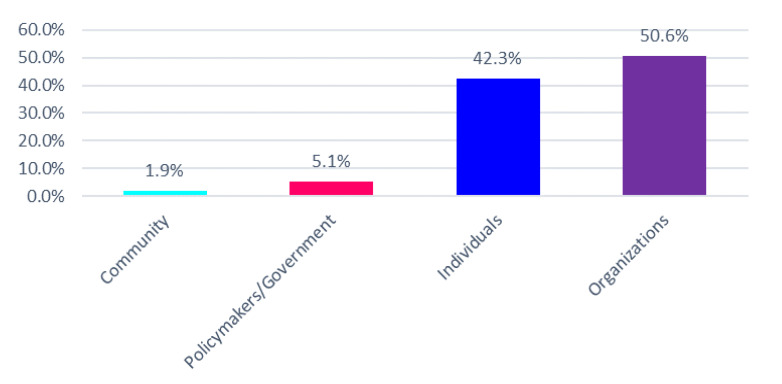
Stakeholder representation: concerns about discrimination in employer-sponsored wellness programs.

**Figure 2:**
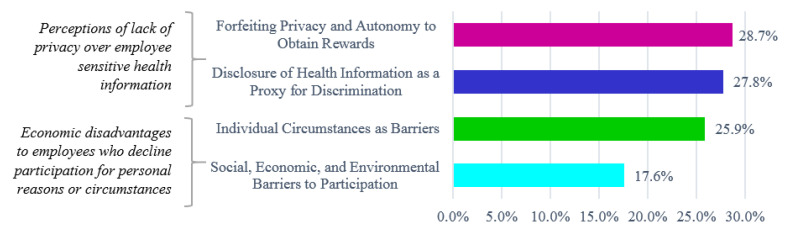
Stakeholder concerns about discrimination in employer-sponsored wellness programs.

Individuals (42.3%) and organizations (50.6%) largely expressed the concerns identified (see Figure 1). A qualitative assessment of public comments in the Federal Register, court case documents, and transcript of public testimony at legislative committee meetings showed that stakeholder concerns fell within two key descriptive themes: (1) perceptions of lack of privacy over sensitive employee health information, and (2) economic disadvantages to employees who decline participation for personal reasons or circumstances (see Figure 2). In addition to reviewing the court case documents that were found through our searches, the following court cases were cited by public stakeholders in the Federal Register and reviewed given their relevance to concerns about discrimination in workplace wellness programs: EEOC v. Flambeau, General Dynamics Land System v. Cline, Havasupai Indians v. Arizona State University, Leonard F. v. Israel Disc. Bank of New York, Ohio Public Employees Retirement System v. Betts, and Seff v. Broward County.

### 3.2. Determining Privacy and Nondiscrimination Best Practices: Interviews with Legislators and Legislative Staff

Semi-structured interviews (in-person and phone) were conducted with 10 US legislators and legislative staff to discuss the concerns identified from the Federal Register, court cases, and video/transcripts of public testimony at legislative hearings. Among those interviewed, 30% were legislative staff, 80% were female, and interviewees represented eight states or territories (Minnesota, California, Florida, New Hampshire, Virgin Islands, Illinois, Missouri, and Pennsylvania). Most of the interviewees were legislators in the US Congress (70%) and half (50%) were over 55 years of age. The majority of interviewees (90%) were Democrat or represented democratic Congressional offices.

A grounded theory approach was also used to conduct inductive coding and a qualitative analysis of 75 comments from interview transcripts [23]. Our analysis revealed two key best practice themes (with 97.3% agreement between the two interview coders), which were promoting a (1) culture of trust and (2) culture of health or wellness in the workplace. We defined “culture of trust” as a “collective norm that embodies the conviction that another person or entity will perform certain actions, or behave as promised”, which we adapted from previous definitions in the social science literature [24,25]. Adapting also from the literature, we defined a “culture of health or wellness” as “an intentional, collective norm to ensure that diverse individuals and populations lead healthier lives now and for generations to come by making it easier and more rewarding for them to select lifestyles that foster health” [26,27,28].

**Figure 3:**
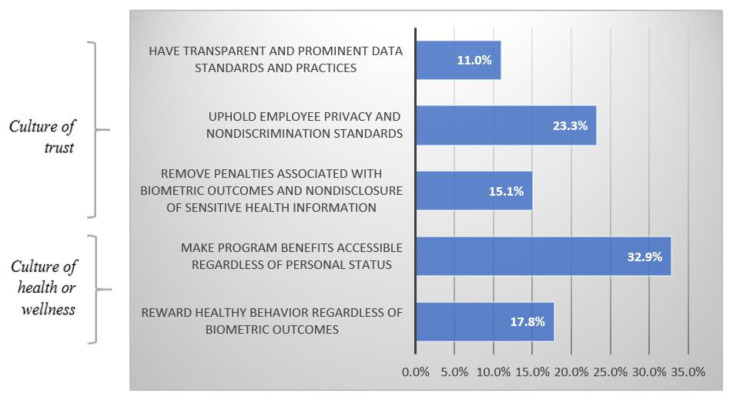
Prevalence of key themes and best practices for employer-sponsored wellness programs.

Among the comments in coder agreement (73/75), best practices within the “culture of trust” theme were: (1) have transparent and prominent data standards and practices, (2) uphold employee privacy and nondiscrimination standards, and (3) remove penalties associated with biometric outcomes and nondisclosure of sensitive health information. Best practices within the “culture of health or wellness” theme were: (1) reward healthy behavior regardless of biometric outcomes and (2) make program benefits accessible regardless of personal status. Figure 3 presents the prevalence of the themes and best practices among the comments extracted, and Table 1 summarizes illuminating interview quotes within those themes and best practices.

## 4. Discussion

Very few, if any, empirical studies elucidate public and policymaker concerns about the privacy and discrimination risks that are inherent to employer-sponsored wellness programs that engage third party companies offering DTC genetic testing services, consumer wearables for wellness tracking, and wellness apps. Moreover, no study to date has been conducted with the dual purposes of conveying public stakeholder concerns about employer-sponsored wellness programs to policymakers and garnering possible best privacy and nondiscrimination practices to address these concerns from the perspectives of policymakers. This study thus offers a novel glimpse into how various stakeholders, including policymakers, perceive employer-sponsored wellness programs as beneficial if privacy and nondiscrimination best practices are both known and enforceable. Additionally, our proposed best practices align with best practice recommendations recently proposed in late empirical work regarding personalized wellness programs [29].

Concerns about employer discrimination based on genetic information are not new to the policy landscape; a controversial 2017 federal House Bill 1313 (HB 1313) sought to grant employers access to employees’ and employee beneficiaries’ genetic health information under the notion of “preserving employee wellness” [16,30,31]. Social justice critics responded by arguing that genetic information privacy is required to deter or prevent the rectification of historical acts of discrimination [32,33,34,35]. Some have also argued that genetic testing cloaked under the stewardship of employer-sponsored wellness programs can reveal to an employer genetic health risks that were not originally sought (secondary results) but bear clinical relevance or importance to employees [36]. There are also concerns that health-contingent (versus participatory) wellness programs are inherently discriminatory because they allow employers to treat employees differently based on health status and assess penalties if employees do not complete a health risk assessment, which would imply coercion in the sense that employees must disclose non-work-related yet sensitive health or genetic information to employers [37,38,39]. Our study confirms that previous concerns expressed by experts through blogs and peer-reviewed commentary actually exist among a broad range of stakeholders. Importantly, this study provides empirical evidence that there are concerns about how the disclosure of sensitive health information to employers and unregulated third parties might lead to information asymmetries, or an imbalance in power through an amassing of information, that might enable employers to engage in possible backdoor employee discrimination.

Employers are well-positioned to inspire a culture of health within the workplace by exploring and implementing innovative strategies that are privacy-centric and non-discriminatory [40]. For example, if leveraging mobile technology, employers can require wellness app vendors to undergo intake processes that require that apps be transparent about whether and how they collect, process, and share employees’ sensitive health information with the employer. Transparency around this will allow employees to be fully informed about possible privacy risks.

Employees should also be able to opt out or opt in without penalties and have alternative options to engage in the workplace wellness program. Alternative options might involve leveraging existing resources within the workplace to offer free or low-cost meals and snacks that employees can take home and healthy cooking classes during work hours [41]. Doing so might help employers overcome the costs associated with wellness program implementation and control for income-level effects on weight loss and wellness program participation [42]. Having alternative options gives both employers and employees flexibility, lowers risk of coercion, and helps employers measure health behavior changes and outcomes following wellness program implementation. Additionally, by rewarding healthy behavior regardless of biometric outcomes, employees can determine, with help from clinicians, biomedical reasons (e.g., genetic causes) for why any intended biometrics outcomes were not achieved.

Limitations to this study were at least three-fold. First, proceedings for related cases settled outside of court (e.g., settled through mediation or arbitration) are not found in in Westlaw, Lexis Nexis, and Bloomberg BNA, and thus, are not represented or reflected in this research. Second, it was inherently difficult to recruit legislators and legislative staff for interviews given the very busy nature of their work generally and during legislative sessions. Therefore, our qualitative interviews were conducted with a relatively small number of, but knowledgeable, individuals. A large interviewee sample size, however, was not the only goal of this qualitative study. Lastly, although snowball sampling assisted with recruitment, it led to a skewed sample interviewee population that was mostly Democrat, causing the qualitative data to insufficiently represent legislative stakeholders from other political parties. The ultimate intent of this study, however, was to identify concerns on which we could base suggested privacy and nondiscrimination best practices. The notion of employer-sponsored wellness programs was supported in general across both political parties. Therefore, there is opportunity for future studies to sample a larger number and/or more representative group of legislators and legislative staff across party lines to identify and examine any differences in opinion.

As consumer genetic testing services grow within the marketplace, privacy and discrimination risks and protections become increasingly blurred across not just a patient-consumer spectrum, but now also an consumer-employee spectrum. Effective policymaking remains slow in comparison to this growing market trend and fails to capture the nuance involved in data exchanges across these spectrums that result in privacy loopholes. In addition to GINA, there is a patchwork of US state laws that, collectively, have an underlying theme of protecting individuals from genetic information discrimination across a wide variety of settings (e.g., housing, banking, employment, insurance, etc.). This patchwork of laws subjects US employers to varying regulation of the novel nature and type of personal data collection under personalized wellness programs.

Other countries such as France, Switzerland, Canada, Australia, and the United Kingdom have also implemented genetic information nondiscrimination protections, although the coverage of these protections ranges from restrictive to laissez-faire [43]. Genetic information privacy and nondiscrimination protections are possible under the EU General Data Protection Regulation (GDPR), a law that offers privacy protection with regard to the processing individuals’ “sensitive data”, as genetic data fall within the GDPR’s special category/definition of sensitive data [44]. The GDPR’s definition or category of sensitive data also includes biometric data, which would include biometric data collected to support an individual’s participation in a wellness program (employer-sponsored or otherwise). The GDPR, however, includes a “public interest” exception to its protections, allowing for the processing of sensitive genetic or biometric data “for reasons of substantial public interest” [44]. Therefore, personalized wellness programs conducted under “strict conditions”, but that (1) involve the collection of sensitive data and (2) are sponsored by public health agencies within the EU, could potentially fall under the GDPR’s public interest exception.

The most novel aspect of this study is the contribution of empirical, qualitative data-driven best practices to support the development and implementation of privacy and nondiscrimination policies and regulations that can be specific or related to the novel practice of personalized wellness programs. A recent survey of 15 personalized wellness program vendors’ websites showed that these vendors were unclear, ambiguous, or not transparent about data sharing with employers [29]. Full implementation of our recommended best practices would fill this transparency gap to hold personalized wellness program vendors and employers more accountable in upholding privacy and nondiscrimination promises to individuals who either participate or have an interest in participating in wellness programs. This is particularly important because, in many employment settings, it is standard practice for employees to undergo standard medical evaluations to assess their readiness for duty or evaluate their degree of disability/impairment [45]. Thus, a fine line exists between promoting wellness in the workplace or using wellness programs as a backdoor strategy to employment redlining based on surreptitious uses of employee or individual health data.

## 5. Conclusions

In the US, employer partnerships with companies that offer DTC genetic testing and/or digital health services (consumer wearables, wellness apps, etc.) for the provision of wellness programs raise privacy concerns because employers and such companies are not regulated by HIPAA yet collect, process, and share raw, or insights gleaned from, sensitive health information. In *AARP v. EEOC*, a federal court rejected wellness program regulations under the ACA, stating that the regulations failed to demonstrate “voluntariness” and meet nondiscrimination standards under the GINA and the ADA. As a result, wellness program regulations under the ACA were vacated in January 2019, with new proposed regulations due out soon. Moreover, in the EU where the GDPR would apply to the collection and use of EU citizens’ consumer health and genetic data, there are potential regulatory loopholes to consider within the GDPR regarding “public interest” exceptions that might expose personalized wellness program participants to certain social risks, should privacy and nondiscrimination best practices not be publicly known, followed, and enforced. Our findings showed that individuals and organizations as public stakeholders and policymakers have unaddressed concerns about employer-sponsored wellness programs that center on (1) perceptions of lack of privacy over employee sensitive health information and (2) economic disadvantages to employees who decline participation for personal reasons or circumstances. Employers, policymakers, and other stakeholders should, therefore, consider and/or implement the key themes and our proposed best practices to ensure that employer-sponsored wellness programs are truly voluntary, accessible regardless of personal status, and nondiscriminatory, and that they promote a culture of trust and wellness in diverse workplaces offering personalized wellness programs.

## Figures and Tables

**Table 1:** Illuminating quotes within key themes and best practices.

**Reward healthy behavior regardless of biometric outcomes (Culture of health or wellness)**
“You know, I think that in life in general, you need skin in the game of whatever. And so, I think that there needs to be incentives and—but people are going to have to have responsibility as well. So, it depends on program-to-program. To speak as a whole, you can’t do that. But I generally believe that you should be rewarded for good behavior.”
“Well, regardless of the evidence, I still think it’s a good thing to do to encourage people to take better care of themselves, and maybe it doesn’t save the company any money to develop these programs, but I still think, again, just for the individual that it’s a good thing to do, because hopefully people will be healthier, and of course you can’t argue with that. That’s better for the individual and their families and those kind of things. So, I mean, I wasn’t aware that there was very little evidence, but I still think it’s a good thing for the person, and it’s a nice thing for the company to offer. You can’t always give more money, but there’s other things you can do like wellness programs or a extra holiday, that kind of thing, so I still think it’s good for the culture of the organization when they talk about what are great places to work.”
**Make program benefits accessible, regardless of personal status (Culture of health or wellness)**
“If you have insurance through your employer, and many people do, a lot of people don’t, so if your employer is providing insurance that’s wonderful. I hope we get to a place where we’re no longer employer-based insurance, but the reality we live in is a lot of people have employer-based insurance. The insurance should be offered, and that’s it. The door closes. The cost match of the insurance is the cost match of the insurance. Then you get into pregnancy, you get into people recovering from surgery. All those things start affecting your ability to participate in a wellness program. You click in, you click off. Who’s going to measure that? What are you going to do? So, the cost of your health insurance should be across the board the same for all employees, and employers should never be able to look into your mental health records, your physical health records.”
“I can see the thing about employees wanting to participate and needing to participate because of the incentive, but I could also see a lot of employees that might work a second job and might have childcare issues and might have a 100 and so reasons why they can’t if it’s after work or if it’s during work. Their jobs might not be good, so, I mean, I think that the best way to do it is to have a wellness program in a workplace that is free, accessible to everybody without going through all of that.”
“I think the employer should provide both the time and the means to participate in the wellness programs. I don’t think they should be required to be completed outside of the work areas-- hours or outside of the work areas so if it involves going to a gym, the gym should be on site and it should be during work hours. I don’t think it should be a burden on the employee.”
“I think that if employers are going to offer a wellness program, then it should be conducive to the workplace and where their employees are. It should not be a burden to employees, and I believe many people probably feel similarly in that respect… So really trying to make it seamless and truly integrated within the workplace would be ideal for a wellness program. Something that could be accomplished in the office or wherever the workplace is and that there’s an understanding between employer and employee that the facilities or the whatever the activities are associated with the program can be done during work hours.”
**Remove penalties associated with biometric outcomes and nondisclosure of sensitive health information (Culture of trust)**
“I think you probably hit it spot-on in terms of running the risk of coercion… obviously employers have a justification for being invested in their employees’ wellness. It makes sense, especially if they’re providing healthcare. At the same time, you really need to ensure through the incentives you’re crafting around these that it truly is a voluntary program and that the employees are protected so that the incentives are not so great that then it flips to be almost punitive if they are not participating, and so it’s definitely finding that balance and attenuating the program to ensure that there are the right guardrails around the program and the employees.”
“Well, I mean, I do think people are responsible for their health, but I think it’s their personal choice, so, again, going back to how wellness programs are set-up, I think it should be set-up in an incentive-type way to encourage people to do it, but if people choose not to do it, I mean, that’s their choice. I don’t think you should make people do it. I think about myself, how I gain and lose the same 10 to 15 pounds every year, and I only lose the weight, but I have my mind really set toward it, and I’m really determined that even if someone pushes me into it if I’m not determined it’s not going to happen, just like people who have a drinking problem or smoking, trying to quit smoking, that they have to first want to do it, and then it’s still hard. And I think someone that’s pregnant, I mean, even though some people that are pregnant still exercise or whatever, but it’s still a choice, so I don’t think people should be penalized.”
**Uphold employee privacy and nondiscrimination standards (Culture of trust)**
“So I think that there’s a careful line that we have to ensure that at a policy level when we look at regulations around employee wellness programs that we don’t cross a line into essentially what would be medical underwriting from an employer standpoint, and, you know, the ACA did a lot in terms of progress for medical underwriting, but now that we’re in this new era of employee wellness and more open data and more accessible data, I think we really need to be mindful that it is being used in the right way and wouldn’t be used in a discriminatory fashion, wouldn’t be used to cherry pick employees in hiring process potentially or promotional process, and that it isn’t, you know, it isn’t used, again, against employees. That it wouldn’t be turned into a punitive measure.”
“You know, of course I believe in respecting one’s privacy, and I think that’s a true balance somewhere in between so that employees are able to keep their privacy but be able to participate in the wellness programs. What the answer is to get there, I think that’s where you have so much debate within the agencies and also within Congress. So-- but I think… for the most part, I believe that you need to have a balance of respecting employees’ privacy and still be able to utilize the programs.”
“…I think that’s a difficult one. I mean, if you have a large pool of employees, potentially if you’re not giving the names of employees but just giving statistics, it might be easier, but, you know, in smaller workplaces, it might become very evident which employee you’re talking about when you’re looking at some of those metrics. Yeah, I think that’s something that the workarounds on that might be a little more difficult.”
**Have transparent and prominent data standards and practices (Culture of trust)**
“…To whom does the health data belong? Is it the company who’s making the assessment or does it belong to the employee or the employer? Is it shared? So, making all of those elements clear, that’s not a very straightforward process right now. I mean, companies don’t have standards that cross across each company, or what have you.”
“Well, I definitely think before an employee gets involved in a wellness program there has to be some type of contractual agreement around the issue of privacy and how the information gets used. Now, of course because of hackings, cybersecurity and those kind of things there’s always a chance that your information can get out, but when you get a credit card there’s that chance also, but I think there has to be agreements set-up in the beginning about how and why that information can be used in the privacy document.”
